# Do magnetic resonance imaging features differ between persons with multiple sclerosis of various races and ethnicities?

**DOI:** 10.3389/fneur.2023.1215774

**Published:** 2023-06-28

**Authors:** Nabeela Nathoo, Burcu Zeydan, Nur Neyal, Cynthia Chelf, Darin T. Okuda, Orhun H. Kantarci

**Affiliations:** ^1^Division of Multiple Sclerosis and Autoimmune Neurology, Department of Neurology, Mayo Clinic, Rochester, MN, United States; ^2^Department of Radiology, Mayo Clinic, Rochester, MN, United States; ^3^Mayo Clinic College of Medicine and Science, Library-Public Services, Mayo Clinic, Rochester, MN, United States; ^4^Department of Neurology, The University of Texas Southwestern Medical Center, Dallas, TX, United States

**Keywords:** African American, ethnicity, Latin American, magnetic resonance imaging, multiple sclerosis, neuroimaging, race

## Abstract

Those of African American or Latin American descent have been demonstrated to have more severe clinical presentations of multiple sclerosis (MS) than non-Latin American White people with MS. Concurrently, radiological burden of disease on magnetic resonance imaging (MRI) in African Americans with MS has also been described as being more aggressive. Here, we review MRI studies in diverse racial and ethnic groups (adult and pediatric) investigating lesion burden, inflammation, neurodegeneration, and imaging response to disease modifying therapy. We also discuss why such disparities may exist beyond biology, and how future studies may provide greater insights into underlying differences.

## 1. Introduction

Historically, it was believed that multiple sclerosis (MS) was more common amongst those who are White which has been challenged in recent years, and, in fact, has been demonstrated to be equally as prevalent in those who are Black as it is in the White population in Southern California (1, 2). Despite similar prevalence, MS clinical course descriptors (relapses or progression), and radiological features (new and/or enhancing lesions), in those that are African American and Latin American are more aggressive compared to those who are non-Latin American White (3–6). Furthermore, Black people with MS (pwMS) have the highest mortality under the age of 55 (7). These findings of disparity raise the question of whether discrepancies are due to individual socioeconomical or biological factors such as; 1) general limitation of access to care, including access to MS specialists due to varying levels of insurance coverage and/or simple lack of facilities, 2) general limitation of access to higher efficacy disease modifying therapy (DMT) choices due to the high cost of DMTs, 3) social norms surrounding health issues including cultural differences on the understanding and implications of illness (8), 4) potential for implicit bias leading to different timelines for diagnosis and use of DMT between populations, along with poor encounters with the medical system deterring future engagement, 5) other complicating medical comorbidities, 6) different genetic-epigenetic-immunogenic risk for prognostic determinants of MS (intensity of relapses, location of lesions, recovery potential and neurodegeneration), or 7) other not yet identified factors. It is most likely, however, that some combination of all of these factors plays a role in the differential disease trajectories that are present due to race and ethnicity. To answer the overreaching question of disease variability due to disparity among different races and ethnic backgrounds of MS patients, very large study constructs are needed.

Such additional investigation may guide more personalized treatment strategies, which is important given those of African American descent have been shown to respond less well to interferons (9), but reasonably well to natalizumab (10), dimethyl fumarate (11), and ocrelizumab (12), both clinically and from a neuroimaging perspective. It has also been shown that African American pwMS repopulate their B-cells more quickly than White pwMS, with 33% of African Americans with MS or neuromyelitis optica (NMO) repleting their B-cells, compared to 0% of White patients with MS or NMO between 6 to 12 months after anti-CD20 therapy (13). One potential explanation for this discrepancy is that healthy Black people have a higher baseline CD19 count than White people (14), and so there may be less depletion of B-cells than anticipated because of starting off with a higher B-cell count to begin with (13, 15). Furthermore, plasmablasts are significantly elevated in African Americans and Latin Americans with MS compared to non-Latin American White pwMS (16). Class switching of B-cell populations has been shown to be associated with increased inflammatory activity in MS (16, 17). Taken together, though the data is relatively sparse in the realm of how race and ethnicity can factor into pathophysiological differences in MS, differences in baseline levels of B-cells and class switching to more inflammatory phenotypes could lead to a more robust humoral response, leading to worse disease in African Americans in particular.

The observations of differential responses to DMTs underline that it is paramount to have greater diversity in clinical trials which is currently lacking. A systematic review of MS phase III clinical trials for DMTs from 1995 until 2020 showed that 37.8% of trials did not report on race or ethnicity at all, and that among those that did report on race, 31.1% percent reported on White pwMS only (18, 19).

Magnetic resonance imaging (MRI) is a primary outcome measure for phase II clinical trials and a key secondary outcome measure in phase III clinical trials. MRI is also critical in diagnosis and monitoring of treatment response in MS and has an advanced use in trying to link clinical presentations with specifics of MS pathogenesis as an intermediate biomarker. With the important role of MRI in various facets of MS, we aimed here to systematically review the literature on MRI features in pwMS of different races and ethnicities and speculate on why MRI differences may exist in these populations, as well as how future studies can help address the factors contributing to differences between different racial and ethnic groups with MS.

## 2. Search strategy

**Figure 1:**
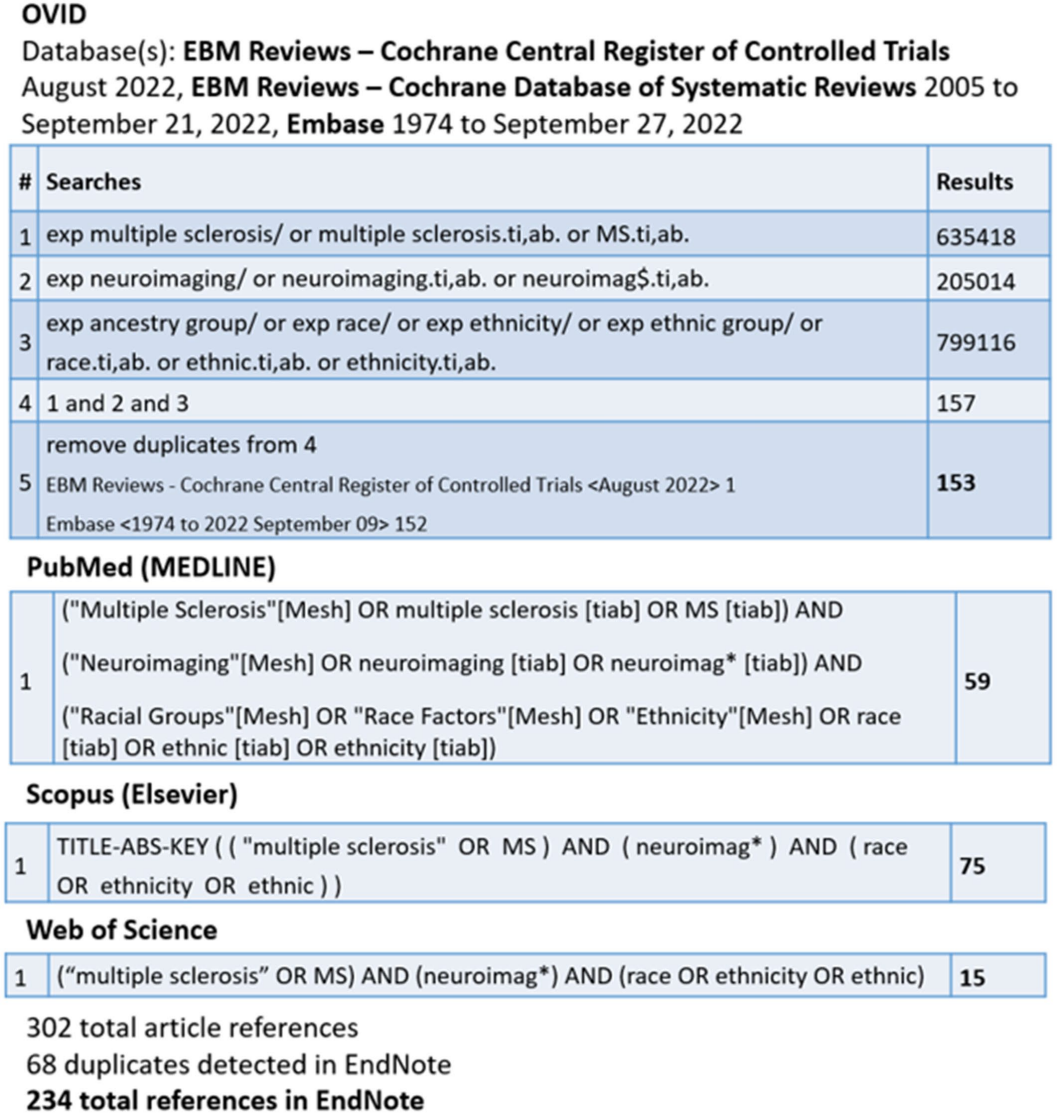
Search strategy.

The search strategy (Figure 1) conducted by C.C. yielded 234 results. Only studies published after 2004 were included given others predated the discovery of aquaporin-4 antibody for NMO.

Abstracts were reviewed by N.Na. to determine applicability to the topic of neuroimaging features in pwMS of different races and ethnicities. Pediatric and adult literature were included. Studies looking solely at other demyelinating disorders, such as NMO and myelin oligodendrocyte glycoprotein antibody-associated disease, were excluded. Similarly, papers with a focus on opticospinal MS (in comparison to more classical MS), where some cases of opticospinal MS could be aquaporin-4 negative NMO, were excluded. We did not include papers focused on applicability and reliability of the Barkhof criteria or McDonald criteria for diagnosing MS in different populations, as the focus of this paper was on the use of MRI for pathogenesis and treatment response. Case studies were excluded, though case series were included. Only studies focused on MRI were included; studies using optical coherence tomography were excluded. In the next step, references within the papers included were also reviewed, and any additional applicable papers that were not captured in the search strategy were obtained and included. Relevant abstracts of all languages were reviewed.

## 3. Details of MRI acquisitions for included studies

The MRI field strength used, acquisition protocols, and analyses methods used for each study included below are outlined in Table 1. The MRI field strength of studies included ranged from 0.35T to 7T.

**Table 1:** MRI acquisition details for studies included.

Study (First author listed)	Racial/Ethnic groups studied	MRI field strength	MRI sequences used	Method(s) of analysis
Pediatric studies				
Chitnis et al. (20)	Non-White vs. White Americans	1.5T	2D double-echo PD 2D T2W SE 2D FLAIR 2D T1W SE (post-contrast) 3D T1W SPGR	Semiautomated lesion segmentation
Waubant et al. (21)	Non-Latin American Whites vs. Others	Pediatric: 1.5T, 3T	T2 FLAIR T1W (post-contrast)	n.p.
		Adult: 1.5T	3D T1W SPGR (pre- and post-contrast) T2W SE	
Weng et al. (22)	Taiwanese	n.p.	T1W (post-contrast) T2W	n.p.
Adult studies				
Absinta et al. (23)	African Americans vs. White Americans	3T and 7T	Precontrast 2D high-resolution gradient dual-echo sequence providing T2* and phase contrasts at 7T Precontrast whole brain 3D segmented EPI sequence providing T2* and phase contrasts at 3T Postcontrast 3D T2 FLAIR at 3T Pre- and post-contrast 3D T1W at 3T and 7T	Automated brain segmentation (Lesion-TOADS) Manual assessment of chronic rim-enhancing lesions
Abuaf et al. (12)	African Americans vs. White Americans	3T	High-resolution 3D T1W 3D or 2D FLAIR	Automated brain segmentation (FreeSurfer cross-sectional image processing pipeline) Automated lesion segmentation (FreeSurfer Samseg pipeline)
Akhtar et al. (24)	Qataris, non-Qatari Arabs, and Asians/others	1.5T	T1W (post-contrast) T2W	n.p.
Al-Kawaz et al. (25)	African Americans vs. White Americans	3T	3D T1W BRAVO (pre- and post-contrast) T2W T2 FLAIR	Automated brain segmentation and cortical thickness measurement (FreeSurfer) Automated regional cortical thickness measurement (FreeSurfer QDEC 1.4) Semiautomated lesion segmentation (Semiautomated lesion mapping method)
Alluqmani et al. (26)	Canadians vs. Saudi Arabians	n.p.	n.p.	Based on radiology reports
Ayele et al. (27)	Ethiopians	0.35T	T2W T2W FLAIR T1W (pre- and post-contrast)	n.p.
Bross et al. (28)	African Americans vs. White Americans	3T	T2W FLAIR 3D T1W MPRAGE T2W TSE	Automated brain segmentation (FreeSurfer) Semiautomated lesion segmentation (Semiautomated edge detection thresholding technique)
Caldito et al. (29)	African Americans vs. White Americans	3T	Multi-slice T2 FLAIR 3D MPRAGE DTI for thalamic segmentation	Automated brain segmentation (MACRUISE) Automated thalamic segmentation (RAFTS) Automated lesion segmentation (Lesion-TOADS) Automated specific lesion segmentation (Subject-Specific Sparse Dictionary)
Cascione et al. (30)	African Americans	n.p.	T1W (post-contrast) T2W	n.p.
Chan et al. (31)	Hong Kong Chinese	n.p.	T1W (post-contrast) T2W	n.p.
Chinea Martinez et al. (32)	Latin Americans	n.p.	T1W (post-contrast) T2W	n.p.
Cree et al. (9)	African Americans vs. White Americans	n.p.	T1W (post-contrast) T2W	n.p.
Cree et al. (10)	Patients of African descent	n.p.	T1W (post-contrast) T2W	n.p.
Deslandes et al. (33)	African descendant Brazilian vs. non-African descendent Brazilian	n.p.	T1W (post-contrast) T2W	n.p.
Gray-Roncal et al. (34)	African Americans vs. White Americans	3T	3D T1W MPRAGE 3D FLAIR	Automated brain and lesion segmentation (MS PATHS Image Evaluation software)
Howard et al. (35)	African Americans vs. White Americans	1.5T	T1W SE (pre- and post-contrast) T2W TSE FLAIR 3D MPRAGE	Automated brain segmentation (SIENAX) Semiautomated lesion segmentation (Semiautomated local thresholding technique)
Kim et al. (36)	Korean	1.5T or 3T	T1W (pre-and post-contrast) T2W FLAIR	n.p.
Kira et al. (37)	Japanese	n.p.	T1W (pre- and post-contrast) T2W PD	Automated brain segmentation (SIENA) Automated lesion segmentation
Mercado et al. (38)	Latin Americans vs. non-Latin American Whites vs. non-Latin American Blacks	n.p.	T1W (post-contrast) T2W	Based on radiology reports
Moog et al. (39)	African Americans vs. White Americans	3T	3D T2W 3D FLAIR 3D high-resolution IR SPGR T1W isotropic	Automated medulla-upper cervical cord segmentation
Nakamura et al. (40)	Japanese vs. Whites	1.5T	T1W SE (pre- and post-contrast) T2W FSE/TSE PD	Automated brain segmentation (SIENAX) Automated thalamus segmentation (FMRIB- Integrated Registration and Segmentation Tool) Semiautomated lesion segmentation (Semiautomated thresholding contour technique)
Okuda et al. (41)	African ancestry vs. European ancestry	3T	3D T2W TSE 3D T2W FLAIR 3D high-resolution MPRAGE T1W isotropic	Automated medulla-upper cervical cord segmentation
Perez et al. (6)	Latin Americans vs. non-Latin American Whites	3T	3D high-resolution T1W MPRAGE 3D FLAIR	Automated brain segmentation (MALF) Manual lesion segmentation
Petracca et al. (42)	African Americans vs. White Americans	3T	Dual echo TSE 3D T1 FFE DIR sequence EPI sequence for fMRI analysis	Automated brain segmentation (SIENAX) Automated WM/GM lesion probability map determination (FMRIB Software Library) Semiautomated lesion segmentation (Semiautomated local thresholding technique)
Saida et al. (43)	Japanese	≥1T	T1W (pre- and post-contrast) Double-echo T2W	n.p.
Saida et al. (44)	Japanese	1.5T	T1W (pre- and post-contrast) T2W PD	Automated brain segmentation (SIENA) Automated lesion segmentation
Seraji-Bozorgzad et al. (45)	African Americans vs. White Americans	3T	T1W (pre- and post-contrast) T2W T2W FLAIR	Automated brain segmentation (SIENAX) Semiautomated lesion segmentation (Semiautomated local thresholding technique)
Shinoda et al. (46)	Japanese	3T	3D DIR	Manual assessment of lesions
Tanaka et al. (47)	Japanese	1.5T	T1W SE (post-contrast)	n.p.
Weinstock-Guttman et al. (48)	African Americans vs. White Americans	1.5T	T1W SE (pre- and post-contrast) Dual T2/PD FSE T2W FLAIR 3D SPGR T1W	Automated brain segmentation (SPM with dilation-based inpainting method) Automated MTR measurement Semiautomated lesion segmentation (Semiautomated edge detection contouring thresholding technique)

2D, 2-dimensional; 3D, 3-dimensional; BRAVO, Brain Volume Imaging; DIR, Double Inversion Recovery; DTI, Diffusion Tensor Imaging; EPI, Echo Planar Imaging; FFE, Fast Field Echo; FLAIR, Fluid attenuated inversion recovery; fMRI, functional MRI; FMRIB, Functional Magnetic Resonance Imaging of the Brain; FSE, Fast Spin Echo; GM, Gray Matter; IR, Inversion Recovery; Lesion-TOADS, Lesion Topology-preserving Anatomy-Driven Segmentation; MACRUISE, Multi-atlas Cortical Reconstruction using Implicit Surface Evolution; MALF, Multi-Atlas Label Fusion; MPRAGE, Magnetization-Prepared Rapid Acquisition Gradient Echo; MRI, Magnetic Resonance Imaging; MS PATHS, Multiple Sclerosis Partners Advancing Technology and Health Solutions; MTR, Magnetization Transfer Ratio; n.p., not provided; PD, Proton Density; RAFTS, Random Forest Thalamus Segmentation; SE, Spin Echo; SIENA, Structural Image Evaluation with Normalization of Atrophy; SIENAX, Structural Image Evaluation with Normalization of Atrophy Cross-sectional; SPGR, Spoiled Gradient-Recalled Echo; SPM, Statistical Parametric Mapping; T1W, T1-Weighted, T2W, T2-Weighted; TSE, Turbo Spin Echo; WM, White matter.

## 4. Pediatric MS studies

### 4.1. Studies on lesion burden, volume, and location

There is a paucity of studies looking at race and ethnic differences in pediatric MS patients in the realm of neuroimaging. One study found that non-White children with MS defined as any of African American, mixed origin/other, and American Indian, had higher T2 lesion volumes and a larger maximal individual T2 lesion volume than White children with MS (20). They did not find differences in the number of T2 lesions or gadolinium-enhancing lesions (20). Though this study included diverse populations, all non-White children with MS were grouped together, which could be problematic given each population in the non-White group may not necessarily behave similarly clinically, or with respect to neuroimaging. This should be accounted for in the study design which is understandably difficult given the lower numbers of pediatric MS patients that could lead to underpowered studies, but is important, nonetheless.

When looking at lesion locations differing between races and ethnicities, pediatric MS patients in Taiwan had typical involvement of the periventricular and subcortical white matter, but also had considerable thalamic and basal ganglia involvement (22). Another study looking at infratentorial involvement found that there was no independent effect of race or ethnicity when comparing non-Latin American White to others, which was how race and ethnicity were delineated in their study (21). They also did not note an effect of race or ethnicity on initial brain MRI with respect to the number of gadolinium-enhancing lesions, total number of T2 lesions, number of large T2 lesions, or location of T2 lesions. However, they did find that non-Latin American White pediatric MS patients had a significantly higher likelihood of corpus callosum involvement (21).

Taken together, the minimal data to date in pediatric MS suggests that racial and ethnic differences may exist with respect to lesion volume and lesion location. However, there are too few studies conducted in this population to date to come to any real conclusions about trends in MRI findings between racial and ethnic groups in pediatric MS. Notably, no studies have been conducted looking at the differences in atrophy in pediatric MS among those of diverse races and ethnicities. There are also no MRI studies looking at treatment response to DMTs in diverse races and ethnic groups of pediatric MS patients. This is important given it is known that African American pediatric MS patients have a higher relapse rate compared to European-origin White pediatric patients living in the United States (49). Thus, it would be useful to see if there is a relationship between this known increase in relapse rate and MRI findings of more aggressive disease, as this could also help dictate DMT selection, which is critical given there are multiple ongoing clinical trials for DMTs in the pediatric MS population.

## 5. Adult MS studies

### 5.1. Studies on lesion burden, volume, and location

Studies looking at lesion burden, volume, and location have primarily included African American and Latin American populations in the United States. Other studies have looked specifically at Japanese MS patients. There are two studies looking at MS in the Middle East. One study was conducted in Ethiopian MS patients.

Across multiple studies, African American patients with MS have been shown to have significantly greater T2 lesion volume compared to White patients with MS (29, 34, 35, 45, 48) with one of these studies determining that this finding remained even after adjusting for age, sex, optic neuritis history, and disease duration (29). In one of these studies, the higher lesion volume in African Americans was due to periventricular and infratentorial lesion volumes being significantly greater than those in White Americans, while juxtacortical and other lesion volumes were not significantly different between groups (34). One study demonstrated that African Americans with MS have a greater number of gadolinium-enhancing lesions compared to White patients with MS (45), indicative of active inflammation. African Americans with MS also have greater T1 lesion volume (35, 48), indicative of severe axonal damage. Few studies have looked specifically at cortical lesions between those of different races and ethnicities, likely in part because other pulse sequences (e.g., double inversion recovery) not part of the standard MS acquisition protocol should be used to optimize detection. One study using 3T MRI that did look at differences in cortical lesion number and volume between African Americans and White Americans with MS found no significant difference between groups with respect to cortical lesion number or volume (42).

With respect to Latin American MS patients, two studies have been conducted. One study found significantly greater T2 lesion volumes in Latin American MS patients compared to non-Latin American White MS patients, and the T2 lesion volumes were correlated with expanded disability status scale (EDSS) scores in Latin American patients (6). Another study looking specifically at Latin American, non-Latin American White, and non-Latin American Black patients in Texas, United States did not find any significant differences between groups with respect to the number of T1, T2, and gadolinium-enhancing lesions at time of diagnosis, nor were there differences between groups in the number of T2 or gadolinium-enhancing lesions involving the spinal cord at diagnosis (38).

Few studies have been conducted in Japanese MS patients. One study compared Japanese MS patients to White MS patients, finding that Japanese patients had significantly larger T2 lesion volume per lesion though no differences in T2 lesion volume in total (40). The T2 lesion volumes had significant correlations with EDSS scores in Japanese patients. With respect to lesion location, Japanese MS patients had lesions in the cerebellum and parietal lobe less frequently than White MS patients (40). Another study looked solely at the number of asymptomatic gadolinium-enhancing lesions in 23 Japanese patients with relapsing–remitting MS (RRMS) due to the presence of gadolinium-enhancing lesions being a pertinent outcome measures in clinical trials; they found that 39% of their cohort had an asymptomatic gadolinium-enhancing lesion with the number of asymptomatic gadolinium-enhancing lesions per scan being 0.37, lower than what has been reported in White MS patients (47). Another study looked specifically at cortical and intracortical lesions in a group of 92 Japanese MS patients using 3T MRI with double inversion recovery (46). This study aimed to relate the presence of cortical lesions with risk alleles, finding that carriers of the HLA-DRB1*1501 had a higher likelihood of and higher number of intracortical lesions than those who were not carriers (46). Interestingly, that study found that those with the HLA-DRB1*0405 allele were less likely to have intracortical lesions (46).

Two studies have looked at MS patients in the Middle East. One was a comparative study, looking at characteristics of RRMS patients in Medina, Saudi Arabia and Edmonton, Canada. More White MS patients in Canada had posterior fossa and spinal cord lesions than Bedouin Arabic MS patients from Saudi Arabia (26). The authors note that the higher propensity for cord lesions in White MS patients in Canada could be confounded by the fact that more spinal cord imaging occurs in Canada (26). The other key factor not addressed in this study was the potential difference in the type and frequency of DMT use in the cohorts studied, which is important because despite the White Canadian MS patients having more infratentorial and spinal cord lesions, EDSS scores were higher in the Saudi Arabian population (26). A different study looked at newly diagnosed MS patients presenting to a tertiary hospital in Doha, Qatar (24). Over half the patients (58%) were Qataris, while 31% were non-Qatari Arabs, with the rest being Asian and classified as “other” (24). Within the Qatari population with follow-up over 36 months, 20% had worsening of EDSS score while radiological disease progression (did not define what progression refers to) was noted in 55% (24). The subgroups of Qataris, non-Qatari Arabs and Asian/other were not compared to know how clinical and radiological activity differed between groups.

There has been one MRI study originating from Africa, in Addis Ababa, Ethiopia. With a 0.35 T MRI scanner, 30 persons with MS were studied, finding classic MRI features of MS with white matter lesions involving the corpus callosum, juxtacortical region, and spinal cord (cervical and thoracic) (27).

Altogether, the clearest trend is that African Americans with MS have greater T2 and gadolinium-enhancing lesion burden compared to White pwMS. Latin Americans with MS have more variable MRI data, which may relate to the fact that they are cited to have a lower incidence of MS compared to Whites with MS (50, 51), making studies potentially underpowered to detect significant differences.

### 5.2. Studies looking at atrophy

Measures of brain atrophy have been shown to correlate better with clinical disability than T2 lesion burden (52–54). Accordingly, studies have been conducted comparing degree of atrophy among those with MS of different races and ethnicities.

Multiple studies have shown that African American pwMS have lower brain volumes than White pwMS with respect to total grey matter volume (34, 45) and cortical thickness (25, 28, 29). One of these studies found an association between reduced grey matter volume and elevated CSF IgG index in African Americans (45). Some studies have also looked at region-specific differences and found that there is greater cerebellar atrophy (42), thalamic atrophy (29, 34) and deep grey matter atrophy (34) in African Americans compared to White Americans. However, one study demonstrated that White Americans had significantly lower thalamic volume compared to African Americans with MS. Notably, this was measured relatively early in the disease course, where patients had an average disease duration of less than 7 years (25). One study also did not find any differences between groups looking at global brain volume (35). Two studies to date have looked at spinal cord volumes. One of these studies found that African American pwMS had greater total spinal cord atrophy, and more specifically, also within the ventral and dorsal regions, compared to White pwMS (39). The other study demonstrated that Europeans with MS had greater volume loss at the medulla-upper cervical spinal cord region than African Americans, but that African Americans had greater baseline pontine volume loss, which continued to worsen at one-year follow-up (41).

Similar relationships have been seen when comparing Latin American pwMS to non-Latin American White pwMS, with significantly lower total brain volume, cortex volume, basal ganglia volume, and lower white matter volumes in Latin American pwMS compared to White pwMS (6). Lower baseline thalamic volume portended a higher risk for progression in the Latin American group (6). Importantly, there were no significant differences between the two groups with respect to demographics (smoking, body mass index, comorbidities, baseline vitamin D levels, educational attainment), age of onset, diagnostic lag, or treatment duration, suggesting that disease is inherently worse in Latin Americans (6). Conversely, a study from Texas, United States comparing non-Latin American White, Latin American, and non-Latin American Black pwMS did not find a significant difference in brain atrophy or spinal cord atrophy between groups (38). However, the number of non-Latin American White pwMS included in this study was low at 11, and thus could have been underpowered to detect differences between groups.

One study compared brain volumes in Japanese MS patients to White MS patients, finding that the Japanese MS patients had significantly lower total brain volume, white matter volume, deep grey matter volume, and thalamic volume compared to White MS patients (40). However, despite the lower volumes, Japanese MS patients had significantly less ambulatory impairment with lower EDSS scores than White MS patients (40). Cognitive and higher cortical function associations were not studied. Medical comorbidities were also unaccounted for. This raises the speculation of how differential reserves of function or spinal cord involvement pattern relate to clinical disability, questions that remain mostly unanswered in studies to date.

The abovementioned study from Ethiopia also looked at atrophy, finding global cortical and corpus callosum atrophy in a minority of persons with MS (27).

### 5.3. Studies using non-conventional MRI metrics

Few studies have used non-conventional “advanced” structural MRI methods. One study using magnetization transfer ratio demonstrated that African Americans with MS had lower magnetization transfer ratio values in lesions, normal-appearing grey matter, and normal-appearing white matter (all in the range of 10–15%) compared to White Americans with MS (48). This would suggest potentially different myelin structure or content between African Americans and White Americans with MS. Another study used both 3 T and 7 T MRI to obtain susceptibility-based MRI sequences including T2* and phase contrast to look at paramagnetic rim lesions in both White Americans and African Americans (23). African Americans were not found to have a statistically greater number of rim lesions compared to White Americans (23). Only one known study to date has been conducted with functional MRI (42). That study used a simple motor task of flexion and extension of the last 4 digits of the right hand, finding that African Americans had decreased activation of the prefrontal cortex compared to White Americans with MS (42). This could be indicative of impaired compensatory activity in African Americans (42).

### 5.4. Studies using MRI as an outcome measure for treatment response to DMTs

Studies have been conducted looking at the effects of various MS DMTs on MRI outcomes in those of diverse racial and ethnic backgrounds (Table 2).

**Table 2:** Effects of various disease modifying therapies for multiple sclerosis on MRIs in those of diverse racial and ethnic groups.

Disease modifying therapy	Race/ethnic group	MRI findings
Interferon	African American vs. White Americans	More new T2 lesions in African Americans at 48 weeks compared to White Americans (9)
	Koreans	Proportion with new or enlarging T2 lesions, gadolinium-enhancing lesions reported to be similar to White pwMS (36)
	Japanese	Proportion with gadolinium-enhancing lesions reported to be similar to White pwMS (43)
	Hong Kong Chinese	No significant difference between group treated with interferon and untreated group for percent with T2 lesions in brainstem, cerebellum, spinal cord (31)
Natalizumab	African Americans	Decreased accumulation of new gadolinium-enhancing and T2 lesions in those treated with natalizumab compared to placebo (10)
	Brazilian cohort comparing those of African descent to those of non-African descent	No significant difference between groups for development of new gadolinium-enhancing or T2 lesions (33)
Fingolimod	Latin American	Lower T2 lesion volumes in those treated with fingolimod compared with interferon or placebo (32)
	African American	No significant differences in T2 lesion volume nor total brain volume in those treated with fingolimod compared to injectable DMT (30)
	Japanese	Significant reduction in development of gadolinium-enhancing lesions with fingolimod treatment versus placebo (37, 44)
Ocrelizumab	Black persons vs. White persons	No significant differences between groups with respect to cortical grey matter thickness; volumes of caudate, putamen, thalamus, brainstem (12)

DMT, disease-modifying therapy; MS, multiple sclerosis; pwMS, people with MS.

#### 5.4.1. Interferons

Variable responses to DMTs between racial and ethnic groups were first demonstrated in an exploratory post-hoc analysis of the European North American Comparative Efficacy (EVIDENCE) study, which compared different formulations of interferon beta-1a (Avonex vs. Rebif). This post-hoc analysis demonstrated that compared to White Americans, African Americans were less likely to remain relapse-free and developed significantly more new T2 lesions on MRI at 48 weeks (9). However, whether the patients who had relapses and new MRI lesion development had neutralizing antibodies to interferons was unknown in this study.

The remainder of studies looking at the use of interferons in non-White pwMS have been conducted in Asian countries. A retrospective study looking at five centers in Korea found that 55% of their study population was free of new or enlarging T2 lesions, and gadolinium-enhancing lesions, reportedly similar to White pwMS. However, a comparative percentage in White pwMS or number of new or enlarging T2 lesions in the Korean population was not provided in this study (36). Similar results were seen in a study of Japanese RRMS patients with those on interferon-beta 1a treated for 6 months, with a significant reduction in the number of gadolinium-enhancing lesions in the pre-treatment versus post-treatment periods with values indeed similar to what has been seen in White pwMS (43). Conversely, there was a study looking at Hong Kong Chinese pwMS where no significant differences were seen in the percent with T2 lesions in the brainstem, cerebellum, and spinal cord between those treated with interferon beta-1a and not, which corresponded clinically with no significant differences in EDSS between groups (31). However, exclusion of NMO was done via aquaporin-4 antibody testing using indirect immunofluorescence, not the contemporary cell-based assay, which begs the question of whether cases of NMO were inadvertently included, which does not respond well to interferons. Testing for neutralizing antibodies to interferons was also not undertaken in this study.

Overall, given the known mechanism of action of interferon in MS, these results would suggest potentially higher disease activity potential in African American MS patients where the relatively lower efficacy DMTs (compared to higher efficacy drugs discussed later) may be insufficiently effective. Alternative explanations as related to immune activation patterns that may be determined by ethnicity can also be more obvious than with higher efficacy DMTs. The results may highlight the reason to consider higher efficacy DMTs as first-line treatment in African American patients. This is further suggested by the studies below.

#### 5.4.2. Natalizumab

Post-hoc analysis was undertaken looking at the African Americans included in the Natalizumab Safety and Efficacy in RRMS (AFFIRM) and Safety and Efficacy of Natalizumab in Combination With Interferon Beta-1a in Patients with RRMS (SENTINEL) studies (10). When comparing those that were treated with natalizumab versus placebo, there was reduced annualized relapse rate (ARR) and accumulation of new MRI lesions over 2 years including both gadolinium-enhancing lesions and T2 lesions in those treated with natalizumab (10). A different study looked at a Brazilian cohort, comparing African descendants to non-African descendants with MS, finding that there was no significant difference between the two groups with respect to development of new gadolinium-enhancing or T2 lesions with natalizumab treatment, with both groups responding well to treatment (33). Altogether, though studies have been minimal, Black pwMS seem to benefit from treatment with natalizumab. The reasons why this is the case are unclear; it could be that natalizumab is a high efficacy medication and would be beneficial, irrespective of race or ethnicity.

#### 5.4.3. Fingolimod

Post-hoc analysis was undertaken for Latin American pwMS for the FTY720 Research Evaluating Effects of Daily Oral therapy in MS (FREEDOMS, FREEDOMS II) studies comparing fingolimod to placebo and Trial Assessing Injectable Interferon vs. FTY720 Oral in RRMS (TRANSFORMS) which compared fingolimod to interferon beta-1a (32). Latin Americans with MS treated with fingolimod had lower T2 lesion volumes compared to those treated with interferon beta-1a or placebo along with reduced ARR for 2 years (32).

Post-hoc analysis of the 141 African Americans with MS included in the Prospective, Randomized, active-controlled, open-label study to Evaluate patient retention on Fingolimod versus approved first-line disease-modifying therapies in adults with Relapsing–remitting MS (PREFERMS) comparing fingolimod to an injectable DMT showed no significant differences between groups with respect to T2 lesion volume or normalized brain volume (30).

A randomized-controlled trial (RCT) was undertaken in Japanese people with RRMS comparing fingolimod at a dose of 0.5 mg or 1.25 mg versus placebo for 6 months, with the primary endpoint being percentages of patients without development of gadolinium-enhancing lesions at follow-up at three and 6 months (44). Fingolimod treatment at either dose was significantly more effective than placebo, with 70–86% not developing gadolinium-enhancing lesions at three and 6 months compared to 40% in the placebo group (44). This work was then extended to 12 months with all participants being put on fingolimod 0.5 mg with maintenance of a high percentage being free of gadolinium-enhancing lesions at months 7–12 (all greater than 80%) (37). Similar proportions were free of new or enlarging T2 lesions (37). There was also a higher proportion of subjects with reduced ARR at months 7–12 compared to months 0–6 (37).

Although clearly effective in the Latin American and Japanese populations with MS, the relative efficacy of fingolimod with respect to MRI outcomes specifically in the African American population is less clear. This is pertinent given the use of not just fingolimod, but the other S1P inhibitors as a DMT with efficacy that is in the middle of the spectrum, which could provide a viable oral option; this would be critical in situations where access to an infusion center is limited.

#### 5.4.4. Ocrelizumab

A retrospective study was conducted on Black and White pwMS on ocrelizumab with MRIs being obtained in a proportion of the study subjects at baseline and year two (12). No significant differences were present between groups comparing cortical grey matter thickness, along with volumes of the thalamus, caudate, putamen, and brainstem (12).

## 6. Discussion

Although the number of studies conducted in the realm of neuroimaging features among diverse racial and ethnic groups with MS remains low, certain trends are apparent. First, African American pwMS consistently have worse MRI findings (higher lesion burden, substantial atrophy) compared to White pwMS. Second, data in Latin American pwMS is more variable which may relate to their lower cited incidence of MS compared to non-Latin American White pwMS (50, 51) as mentioned above, but may also relate to underdiagnosis in certain Latin American countries (50). Third, though studies have been minimal in Asian populations, it appears that Japanese pwMS may have higher MRI burden though can have lower EDSS and respond well to DMTs. Part of this could pertain to the relatively lower number of lesions in the cerebellum compared to those who are White in one study (40), but otherwise why this is the case is unclear, and if this relationship will hold true when higher numbers of patients are studied is uncertain. Comparing the presence of lesions specifically in the brainstem and spinal cord in Japanese versus White pwMS could also be useful as these tend to lead to greater clinical disability which could translate to a higher EDSS.

### 6.1. DMTs: response to treatment, discontinuation of treatment, and access to treatment

In the realm of treatment, there is a pressing need for studies using DMTs in diverse racial and ethnic groups with MS with MRI as an outcome measure, given different responses to treatment, particularly in the African American population. As we regularly factor the presence of medical comorbidities into our DMT selection for MS patients, so too should racial or ethnic group based on data we have to date (e.g., perhaps avoiding interferons in African Americans and instead opt for dimethyl fumarate, natalizumab, or ocrelizumab). Given the increasing number of anti-CD20 therapies coming down the pipeline and their high frequency of use, we need more data on how responses to treatment varies between racially and ethnically diverse pwMS. There has been speculation that elevated CSF IgG index in African Americans with MS could signal that use of anti-CD20 therapies is indicated, given its effect on the humoral immune system (55). Notably, there are no known studies using certain oral options including teriflunomide, siponimod, ozanimod, or cladribine. Dimethyl fumarate has shown clinical efficacy in African Americans with MS though MRI data was unavailable in that study (11).

Along with looking at treatment efficacy, data is needed on the safety profile of these agents in diverse populations. Although higher efficacy treatments seem to be beneficial in African American pwMS, painting all patients of a particular racial or ethnic group with the same brush can be dangerous, as higher efficacy treatments come with more potentially severe adverse events, including death, albeit rarely.

Furthermore, discontinuation of DMTs is an important factor to consider and reasons for this are diverse. One study showed that African Americans or other Black pwMS changed DMT due to injection fatigue (with desire for an oral medication) and subjective perceived lack of efficacy more frequently than White pwMS, but that they were less likely to switch DMT based on side effects (56). These are pertinent issues to keep in mind when pondering different clinical MS trajectories which could, in part, relate to persistence of staying on a specific DMT and the reasons why some pwMS may not stay on a prescribed DMT. Furthermore, the magnitude of DMT waste in this study was over one million dollars in 1 month, with the majority switching therapies for reasons other than inadequate disease control and intolerability (56). This raises the question of whether these DMTs being wasted could be used in populations who have inadequate access to DMTs due to difficulties with insurance coverage. All of this comes around to potentially impacting MRI outcomes from an access issue rather than direct biological differences in various ethnic groups.

Lastly, access to physicians and DMTs vary by region and not by ethnicity alone. Not all DMTs are readily available in every country, with studies from South Africa (57), Sudan (58), and Zambia (59) citing this issue, yet there is increasing knowledge that MS is present in populations in most countries worldwide. It is essential to find accessible and effective treatments that can be utilized in the international population with MS. Otherwise, comparisons are difficult to make between different populations. One way to get around this is in migrant populations who have moved from low access to higher access areas and get diagnosed with MS afterwards. In the absence of elapsed “generational” time for exposure, most differences will be driven by the genetic background. These types of migration studies were done in MS originally, but not necessarily with race and ethnicity stratification.

### 6.2. Possible reasons for variable MRI findings in those of diverse races and ethnicities with MS: pathophysiology

**Figure 2:**
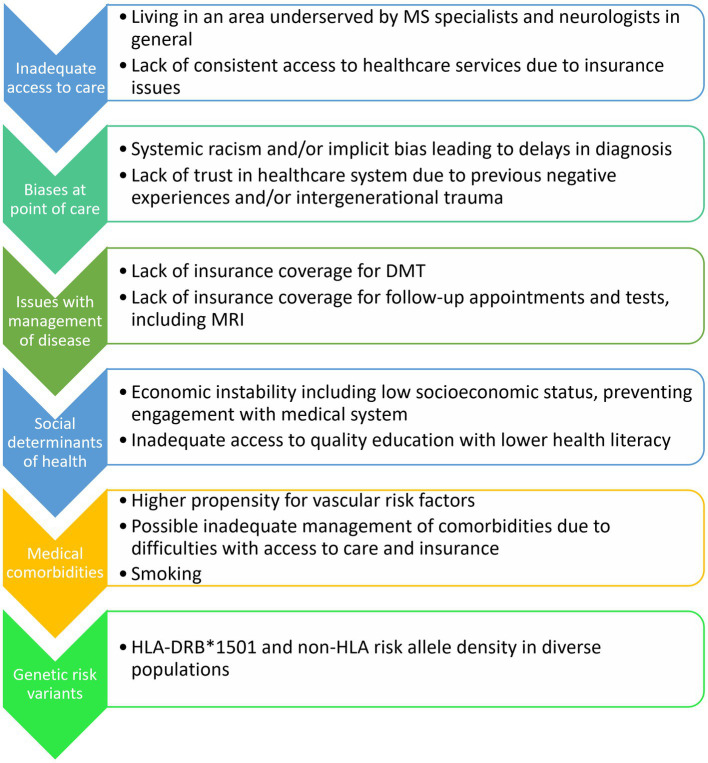
Potential factors contributing to MRI and clinical differences in diverse racial and ethnic groups with MS.

The reasons underlying the findings of different MRI features in those of different races and ethnicities of pwMS remain understudied. Moving forward, rather than simply outlining the MRI features seen between populations, future studies should also aim to elucidate what factors could be contributing to these differences, which few studies have done. Such factors include access to care, social determinants of health, cultural factors, systemic racism and implicit bias, genetic risk variants, presence of cardiovascular comorbidities, smoking status, Epstein–Barr virus (EBV) exposure, and vitamin D deficiency, amongst others (Figure 2). With respect to causal factors for MS, there is a differential effect of EBV on MS in diverse populations. Black and Latin American people have higher Epstein–Barr nuclear antigen-1 (EBNA-1) titers and frequency of seropositivity compared to White people (60), which is relevant given the greatly increased risk of developing MS in those with EBV infection (61). Having a history of infectious mononucleosis due to EBV in Black people leads to an odds ratio (OR) of 4.43 of developing MS or clinically isolated syndrome (CIS) compared to Latin Americans with an OR of 3.36, both greater than in White people with an OR of 2.24 for developing MS (60). EBV infection causes long-term changes in the host cytokine response and prolongs the survival of memory B-cells which could drive inflammation (62). Interestingly, having cytomegalovirus (CMV) positivity was associated with a lower risk of CIS and MS in Latin Americans, even when accounting for HLA-DRB*1501 status and EBNA-1 positivity (60).

Another risk factor associated with development of MS is vitamin D deficiency. African Americans have lower vitamin D levels than non-Latin American Whites and Mexican Americans in the general population (63). This is partially due to differences in melanin levels in the skin, with darker pigmented skin limiting cutaneous vitamin D synthesis, and requiring longer UV exposure to produce the same amount of vitamin D. African American pwMS have lower vitamin D levels than controls based on differences in climate and geography (64). One study showed that having lower vitamin D levels is associated with attack severity with optic neuritis in pwMS (65) while another did not find a relationship between vitamin D deficiency and disease severity (64). Taken together, the predisposition of African Americans to vitamin D deficiency could lead to development of MS and more severe clinical disease. The relationship between vitamin D deficiency and MRI outcomes in diverse races and ethnic populations with MS warrants further exploration.

### 6.3. Possible reasons for variable MRI findings in those of diverse races and ethnicities with MS: social determinants of health

Social determinants of health include both individual and structural factors encompassing five domains: economic stability, education, health and health care, neighborhood and built environment, and social and community context (66, 67). A review has demonstrated that Black pwMS and Latin American pwMS have issues in all of these areas compared to non-Latin American White pwMS including lower income, lower educational attainment, lower health literacy, and higher unemployment rates, all of which could contribute to a worse MS prognosis in Black and Latin American pwMS (66). Being of a lower socioeconomic status can preclude access to higher efficacy DMT (67). These inequities in social determinants of health can also stem from systemic racism and other forms of discrimination, which invariably impact Black and Latin American pwMS a disproportionate amount and can lead to increased mortality (66, 68). Along with this comes added complexity of previous poor encounters with the medical system with potential for intergenerational trauma, which can deter accessing care.

The creation of a National African Americans with MS Registry (NAAMSR) in September 2020 is aimed at determining the impact of social determinants of health on access to care, initiation of DMT, and long-term health outcomes in 20,000–30,000 African Americans with MS (8); this will be an invaluable addition to our knowledge on the impacts of social determinants of health on African Americans with MS at a much larger scale than any data we have at present. It would be of interest to look at MRIs of some of those in this registry to see how T2 lesion burden, gadolinium-enhancing lesion burden, and brain and spinal cord volumes change over time, and how this relates to clinical trajectory and DMT use.

### 6.4. Possible reasons for variable MRI findings in those of diverse races and ethnicities with MS: access to care

Issues with access to care have been well-described in certain populations with MS. African Americans are less likely to seek MS care from a neurologist, though were also more likely to be disabled, which was a risk factor for not seeking care from a neurologist (69). The specific reasons other than disability as to why African Americans were less likely to seek care from a neurologist were not discussed further. One could speculate that the known high cost of DMTs, need to access an infusion center (if using an anti-CD20 therapy like ocrelizumab), and perhaps general lack of connection with a provider of a different racial and ethnic background could factor in.

### 6.5. Possible reasons for variable MRI findings in those of diverse races and ethnicities with MS: genetic risk

With respect to genetic risk, African Americans with the HLA-DRB*1501 risk allele are three times more likely to develop MS than those with the African haplotype (70) and Japanese MS patients with the HLA-DRB*1501 risk allele are more likely to have intracortical lesions associated with more severe disease (46).

### 6.6. How to increase MRI research in diverse racial and ethnic groups with MS

An additional noteworthy issue is access to MRI scanners. There are an average of 0.7 MRI scanners per million people in Africa compared to 40 MRI scanners per million people in the United States (71). In areas where studies are being undertaken that have poor access to MRIs (including both in North America and beyond), low-field portable MRI could be considered, which can detect white matter lesions in MS (72, 73), though this would not necessarily help address the issue of cost. Low-field MRI (0.35T) has been used to aid in diagnosis and management of MS in Ethiopia (27). Upon procuring or building low-field MRIs in low- and middle-income countries, additional considerations come to mind. The first is that the MRI system needs to be functional; in nearly 40% of cases, low-field MRI scanners in Africa were obsolete systems (74). A second aspect is the need for software that is open source to enable better understanding of how to use and repair the MRI (71). A third consideration is how to carry out image analysis which has been proposed to be helped with the use of artificial intelligence (71). In the field of MS, this could relate to automated detection of white matter lesions and measuring brain volume. Lastly, with MRI scanners, trained personnel are needed to operate the MRI scanner, perform necessary maintenance, and to analyze the MRIs. In 2019, the Consortium for Advancement of MRI Education and Research in Africa (CAMERA) was created and assessed MRI use in sub-Saharan Africa, identifying gaps not just in acquisition of MRI scanners, but also the abovementioned issues about maintenance and personnel as well (71, 74). In order to build capacity and sustainable use of MRI in low- and middle-income countries worldwide, high-income countries will need to invest in this, which can also be aided by international imaging societies.

Even if access to MRIs is obtained, though low field can be used, many novel imaging biomarkers require more advanced imaging techniques (e.g., central vein sign, which requires a field strength >1.5T). Therefore, development of practical, easy to apply, and affordable imaging metrics to limit misdiagnosis and monitor treatment efficacy and disability is important to overcome race diversity differences. One example would be the use of manual C5 level spinal cord area measurement as a practical biomarker of disease progression (75). Increasing awareness of and access to such imaging methods should be prioritized.

## 7. Conclusions and future directions

The current review highlights that differences in MRI findings in diverse racial and ethnic groups with MS may be offering clues in this area, may be offering clues to not only the biology of MS but also providing a potential roadmap of personalizing treatment of MS patients with ethnicity being a consideration in addition to age, sex, efficacy, safety profile, access to health care and cost effectiveness. Even with race being a social construct, the reality is that there are differences present between these groups which need to be addressed to provide more equitable care. Ultimately, we should aim to diagnose and manage all of our MS patients well, regardless of race or ethnicity, with a personalized approach and aim for equity in MS care, which necessitates further inquiry into why differences between those of diverse races and ethnicities with MS exist, with MRI providing a key tool for investigation. To help increase MRI studies in those of diverse racial and ethnic groups with MS, there needs to be better access to MRI scanners worldwide, and use of scanners, ideally with software and analysis methods that are open access. In addition to international imaging societies, international MS interest committees (e.g., Latin American Committee for Treatment and Research in MS [LACTRIMS] and Middle East North Africa Committee for Treatment and Research in MS [MENACRTRIMS]) can be part of the effort to acquire and operationalize regular use of MRI scanners to acquire data on diverse populations, given seemingly poorer outcomes in non-White persons with MS, both clinically and radiologically. With improved access and use of MRIs, studies can look at the relationship between imaging findings and the relative contributions of medical comorbidities, inadequate access to care, social determinants of health, cultural factors, systemic racism, and genetic risk variants, all of which warrant further exploration in future studies to help fill in the gaps of why such imaging differences exist.

## Author contributions

NNa, BZ, DO, and OK contributed to the conception and design of the study. NNa wrote the first draft of the manuscript. NNe contributed to the generation of content for figures and tables. CC aided in acquisition of the data and generation of content for figures. All authors contributed to the article and approved the submitted version.

## Conflict of interest

DO received personal compensation for consulting and advisory services from Alexion, Biogen, Celgene/Bristol Myers Squibb, EMD Serono, Genentech, Genzyme, Janssen Pharmaceuticals, Novartis, Osmotica Pharmaceuticals, RVL Pharmaceuticals, Inc., TG Therapeutics, and research support from Biogen, Novartis, and EMD Serono/Merck. DO has issued national and international patents along with pending patents related to other developed technologies. DO received royalties for intellectual property licensed by The Board of Regents of The University of Texas System. BZ receives research funding from the NIH (U54 AG044170) and is supported by the Mayo Clinic Eugene and Marcia Applebaum Award.

The remaining authors declare that the research was conducted in the absence of any commercial or financial relationships that could be construed as a potential conflict of interest.

## Publisher’s note

All claims expressed in this article are solely those of the authors and do not necessarily represent those of their affiliated organizations, or those of the publisher, the editors and the reviewers. Any product that may be evaluated in this article, or claim that may be made by its manufacturer, is not guaranteed or endorsed by the publisher.
